# The scope of carer effects and their inclusion in decision-making: a UK-based Delphi study

**DOI:** 10.1186/s12913-021-06742-4

**Published:** 2021-07-29

**Authors:** Hareth Al-Janabi, Nikolaos Efstathiou, Carol McLoughlin, Melanie Calvert, Jan Oyebode

**Affiliations:** 1 Health Economics Unit, Institute of Applied Health Research, University of Birmingham, B15 2TT Edgbaston, Birmingham, UK; 2 School of Nursing, Institute of Clinical Sciences, University of Birmingham, Birmingham, UK; 3 Centre for Patient Reported Outcomes Research, Institute of Applied Health Research, University of Birmingham, B15 2TT Edgbaston, Birmingham, UK; 4 NIHR Birmingham Biomedical Research Centre, NIHR Surgical Reconstruction and Microbiology Research Centre and NIHR Applied Research Centre, West Midlands, University Hospitals Birmingham NHS Foundation Trust and University of Birmingham, B15 2TT Edgbaston, Birmingham, UK; 5 Birmingham Health Partners Centre for Regulatory Science and Innovation, University Hospitals, University of Birmingham, B15 2TT Edgbaston, Birmingham, UK; 6 Centre for Applied Dementia Studies, University of Bradford, Bradford, Richmond Rd, BD7 1DP Bradford, UK

**Keywords:** Informal care, Economic evaluation, Delphi, Mental health, dementia, Stroke

## Abstract

**Background and objective:**

Health and social care may affect unpaid (family) carers’ health and wellbeing in addition to patients’ lives. It is recommended that such impacts (carer effects) are considered in decision-making. However, the scope of carer effects and range of decisions where carer effects should be considered is uncertain. This study aimed to identify: (i) how different categories of healthcare and social care were perceived to impact on unpaid carers; and (ii) whether there was consensus about when carer effects should be formally considered in decision-making contexts.

**Methods:**

A two round, online Delphi study was conducted with 65 UK-based participants (unpaid carers, care professionals, and researchers) with expertise in dementia, mental health, and stroke. Participants considered two broad forms of ‘interventions’ (patient treatment and replacement care) and two broad forms of ‘organisational change’ (staffing and changes in timing/location of care). Participants assessed the likely impacts of these on unpaid carers and whether impacts should be considered in decision-making.

**Results:**

Participants predicted interventions and organisational changes would impact on multiple domains of unpaid carers’ lives, with ‘emotional health’ the most likely outcome to be affected. Patient treatment and replacement care services (‘interventions’) were associated with positive impacts across all domains. Conversely, timing/location changes and staffing changes (‘organisational changes’) were perceived to have mixed and negative impacts. There was widespread support (80–81 %) for considering carer effects in research studies, funding decisions, and patient decision-making.

**Conclusions:**

This study highlights a perception that carer effects are widespread and important to consider in economic evaluation and decision-making. It highlights the particular need to measure and value effects on carers’ emotional health and the need to use a societal perspective to avoid cost shifting to unpaid carers when introducing interventions and making organisational changes.

**Supplementary Information:**

The online version contains supplementary material available at 10.1186/s12913-021-06742-4.

## Background

Unpaid (family) carers play a vital role in supporting the health and wellbeing of individuals with a disability or illness. This care often comes at the expense of unpaid carers’ own wellbeing, with negative effects for carers’ physical and emotional health, finances, and social activity [1–5]. Carers’ wellbeing may also be affected by the organisation and delivery of patient services [6]. This includes social care (i.e. practical support with everyday tasks such as personal care, for people who have extra needs due to illness or disability) as well as health care. Clinical research has provided a wealth of information about the way in which treatment and care affects patient outcomes. However, we know very little about the effect of patient services on unpaid carers’ outcomes (‘carer effects’).

One area of research where carer effects are particularly pertinent is economic evaluation. Economic evaluations provide a means of systematically evaluating the costs and benefits of new services. Carer effects are important to consider in order to measure and value the full health and wellbeing impacts of patient services on society [7]. Failure to consider carer effects means the economic evaluation is incomplete and may provide misleading information on the impact of a service on societal health or wellbeing. The importance of including carer effects is explicitly highlighted in influential methodological guidelines for economic evaluation from, for example, National Institute of Health and Care Excellence in the UK [8], the US panel on cost-effectiveness [9], and Zorginstituut in the Netherlands [10].

To date, carer effects are still rarely considered in economic evaluation. A review of the economic evaluation literature to 2010 only identified 20 economic evaluations of patient interventions that considered informal (unpaid) care [11]. A more recent study showed carer effects were still neglected, even in areas where unpaid carers are intrinsically involved, like Parkinson’s Disease or Rheumatoid Arthritis [12]. Evidence does suggest that in some areas, such as dementia [12] or paediatric care [13], unpaid care is regularly considered on the cost side when economic evaluations are conducted from a societal perspective. For example, in a UK study of antidepressants in dementia, Romeo and colleagues [14] assess whether the intervention affects the number of hours of unpaid carer time (alongside health and social care resource use). Carer time value is then valued using the opportunity cost method and considered as part of the overall cost, taking a wider payer and carer perspective.

However, it is still the exception rather than the rule to value carer quality of life outcomes within an economic evaluation. Indeed a recent review of NICE appraisals found only 16 of 422 appraisals considered carer outcomes [15]. One highlighted area where more information was needed was *“…unpaid/carer health outcomes of [National Health Service] interventions [and across] disease areas…”.*

Economic evaluation is intended to be an aid to healthcare decision-making – ultimately informing and guiding the services that are provided in society [16]. There is therefore the additional question of whether carer wellbeing should be routinely considered more broadly in decisions about health and care delivery. This includes decisions about the availability of services at a national level and the provision of services to individual patients. Certain government policies, such as the Triangle of Care in the UK [17], and the implementation of mental health services in Australia [18] clearly highlight an important role for unpaid carers in decisions about care provision. However, the degree to which carer wellbeing ought to be considered routinely, alongside patient wellbeing, is open to debate.

The aim of the present study was to identify: (i) how different categories of health and social care were perceived to impact on unpaid carers (‘carer effects’); and (ii) whether there was consensus about when such effects should be considered in decision-making contexts.

## Methods

A Delphi study [19, 20] was used to elicit expert judgements about the scope of carer effects and their inclusion in decision-making. The Delphi method provides a framework for transforming individual opinions into a group consensus [20]. Participants are surveyed remotely, quasi-anonymously, and at multiple time points. Delphi studies have been widely used in healthcare research more generally [21–24] and specifically in health economics to determine quality checklists [25], core resource use items to measure in economic evaluation [26], and evidence needs for public health decisions [26]. Methodological guidance on the Delphi technique has been developed [20, 27] but the approach should be seen as pragmatic and flexible, to meet the needs of the study [28, 29]. In our study, a modified Delphi approach was used, whereby we planned for up to three online rounds, but could end it earlier if a high degree of consensus was reached.

### Sampling

Three types of experts were identified for the Delphi study: ‘unpaid carers’, ‘care professionals’. Unpaid carers were defined as individuals who provided care or support for a family member, friend, or neighbour, due to their illness, old-age, or disability. Care professionals were employed staff working for health and social care organisations that had some experience of the impact of care and treatment on patient and carers’ lives. Research professionals were individuals involved in academic interventional research in one of the three clinical areas. Each group brought different insights on unpaid carer wellbeing and healthcare decision-making.

Three clinical areas were considered: dementia, mental health, and stroke. In all areas, unpaid care is important, but carers face different challenges in relation to the illness, service availability, and their caring role [30, 31]. Our target was to recruit at least 20 unpaid carers, 20 care professionals and 20 researchers across the three clinical areas. This sample size was consistent with previous Delphi studies [28, 32] where the aim is to establish group consensus.

Unpaid carers and care professionals were identified initially through local charities, NHS trusts, and service contacts from the lay panel supporting the research programme. Participants had been invited to take part in prior qualitative interviews and focus groups to establish the mechanisms by which health and social care delivery affected carer wellbeing [33]. This sample was supplemented with a small number of carers who had taken part in a nationwide survey on quality of life [34]. A purposeful sampling strategy [35] was used to ensure diversity in terms of caring role (carers) and job role (professionals). Researcher participants were identified with the assistance of the project advisory group. In total, 124 individuals were invited in March 2018 to participate.

### Online survey

The first round of the Delphi study consisted of an online survey to identify likely carer effects of health and social care. Four broad categories of health and social care were identified based on prior work on mechanisms behind carer wellbeing [33] and input from the lived experience advisory panel [LEAP] [36]. These categories were:

- ***Patient treatments (e.g. medication, psychological support).*** These could affect carer wellbeing, by improving patient outcomes and therefore indirectly reducing the emotional and physical strain on carers.
- ***Services to replace or supplement unpaid care (e.g. formal social care)***. These could affect carer wellbeing by directly reducing the caring load although their use may be linked with guilt or financial expense.
- ***Organisational changes to the timing and/or location of care.*** These can affect carer wellbeing when services become easier (or more difficult) to physically access and combine with daily life.
- ***Organisation changes to staffing.*** These can affect carer wellbeing by changing how well-informed carers feel and their sense of alienation.

The first-round survey consisted of two main sections: (i) Part A, which elicited judgements on the likely impact of service changes on carers, based on participants’ own experiences; and (ii) Part B, which elicited judgements on whether these impacts should be explicitly considered in decision-making. The LEAP provided input on the survey length, language and content, as well as the use of rating scales to record participants’ responses [35].

For Part A, participants were asked whether each of the four categories of health and social care (treatment, replacement, timing/location, staffing) would, on balance, have ‘positive’, ‘negative’, ‘positive and negative (mixed)’ or ‘no’ impact on each of five domains of carers’ lives. The five domains covered mental and physical health effects and resource consequences (personal finances, paid work, free time) highlighted in the literature on carer impact [37, 38] and relevant to economic evaluation [16]. For Part B, participants were asked to what extent they agreed or disagreed (on a six-point scale, strongly disagree to strongly agree) with considering carer effects in each of three decision-making contexts (research, funding, patient care). See Table 1 for a summary. The survey had a total of 16 questions and was delivered using Smart Survey software [www.smartsurvey.co.uk]. Please see Appendix 1 for full survey.

**Table 1:** Content of the Delphi survey

Service changes	Domains of carer impact	Decision-making contexts
***1. Patient treatment (e.g. medication, psychological support)***. This is linked to the ‘patient outcomes’ and ‘compliance’ mechanisms. ***2. Services to replace or supplement unpaid care (e.g. formal social care)***. This is linked to the ‘management of care’ mechanism. ***3. Organisational changes to the timing and/or location of care.*** This is linked to the ‘timing and location’ mechanism. ***4. Organisation changes to staffing.*** This is linked to the ‘information’ and ‘alienation’ mechanisms.	Emotional health Physical health Finances Paid work Free time	A. **Research** on these interventions should include finding out how they affect carers’ lives. B. Carer impacts should be considered in **funding decisions**. C. Carer impacts should be considered by professionals in **decisions about patient care**.

In the first round, potential participants were sent a survey link and an information sheet. They were given two weeks to respond and were reminded that their response was voluntary. Non-responders were reminded a day before and three days after the deadline. Data were summarised by participant role (carers, care professionals, researchers) and used to create individual feedback sheets to be used in the second round (see Appendix 2). The feedback sheets reported the proportion in each participant role (and disease area) that agreed/disagreed that carer effects should be considered in the 12 different decisions. Participants’ response to the sets of question below were summarised by role and disease area to generate frequencies relating to:

- the domains of carers’ lives affected by service delivery;
- whether such effects would be positive, negative, mixed, or absent.

Consensus was studied in relation to the strength of agreement that carer effects should be considered for different types of service, decision-making context, and disease. Consensus was defined as at least 70 % agreement [20, 29, 32] in the top third (i.e. strong/moderate agreement) of the scale.

In the second round, all responders were sent a follow-up survey two months later by email. Responders were separated into the three health conditions. This survey contained the ‘Part B’ questions and a ranking question, requiring respondents to identify the *most* important decision context and category of health and social care for considering carer impacts. This was introduced to encourage respondents to prioritise contexts for collecting and using data on carer effects. Individual feedback sheets were also provided for participants, with their own responses and the aggregate sample responses to the ‘agree-disagree’ questions. Participants were asked to consider first round responses in their decision (see Table 2 below) and were assured that they did not have to conform to the group view. Quasi-anonymity [20] of the participants was maintained throughout, with the participants unaware of each other’s identities. A third online round of the Delphi was not conducted as consensus was reached.

**Table 2:** Text at the beginning of round 2

“The aim of round 2 is to see if a consensus emerges on the circumstances when impacts on unpaid carers should be taken into account, alongside the service user, in decision-making. This document presents your answers alongside the answers of the rest of the 20 respondents with expertise in dementia care. We would like you to consider all the responses and judge whether you wish to stand by or change your view. Either is fine! Please review the document as you re-complete the online survey.”	

## Results

Round 1 of the Delphi survey was completed by 65 of the invited individuals (52 %), with 59 of these individuals (91 %) completing round 2 of the survey. The characteristics of the baseline sample are shown in Table 3.

**Table 3:** Characteristics of the Delphi study participants (*n*=65)

**Characteristic**	**N (%)**
**Primary clinical area**	
Dementia	21 (32%)
Mental health	21 (32%)
Stroke	23 (35%)
**Participant’s primary role**	
Unpaid carer	21 (32%)
Researcher	23 (35%)
Care professional	21 (32%)
**Experience in role**	
** >**10 years	41 (63%)
**Experience of service**	
Treatment	54 (83%)
Replacement care	44 (68%)
Timing/location change	35 (54%)
Staffing change	40 (62%)

### Perceived impacts of service changes on unpaid carers

**Fig. 1:**
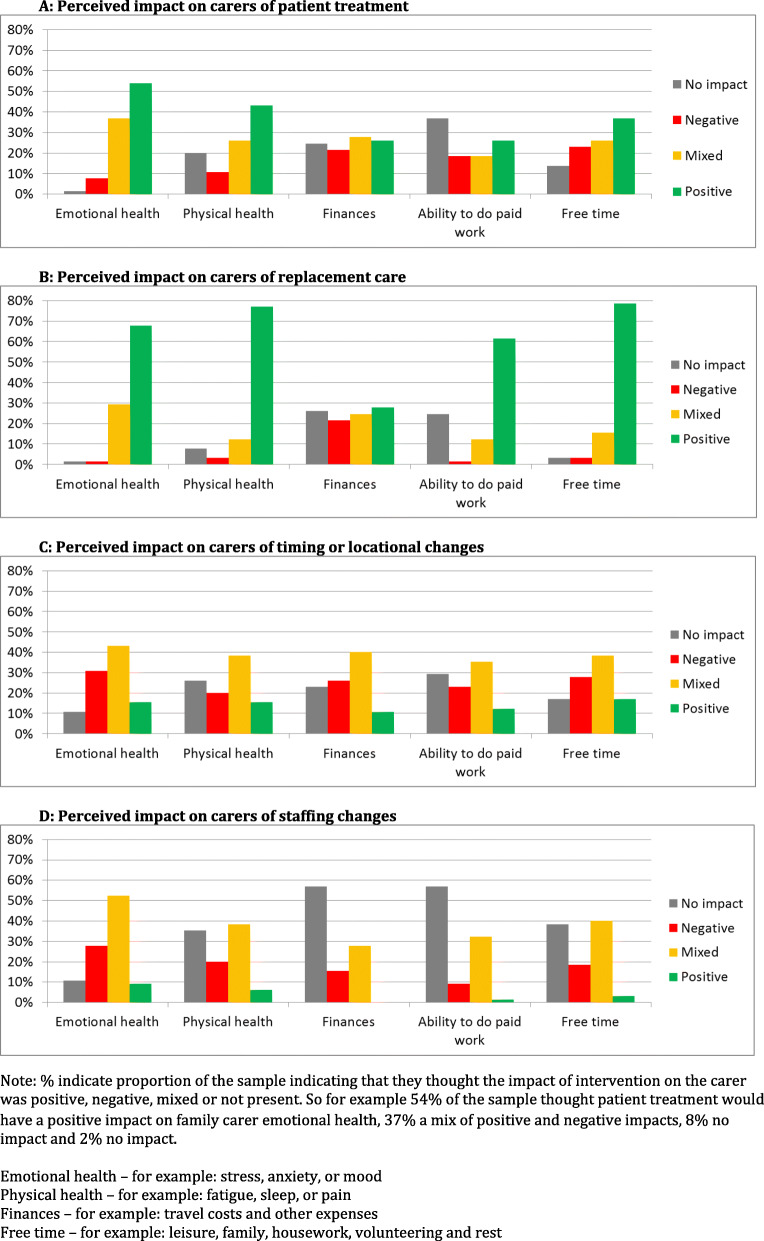
Perceived impact on carers of different aspects of health and social care (*n* = 65). Perceived impact on carers of replacement care. Perceived impact on carers of timing or locational changes. Perceived impact on carers of staffing changes. Note: % indicate proportion of the sample indicating that they thought the impact of intervention on the carer was positive, negative, mixed or not present. So for example 54% of the sample thought patient treatment would have a positive impact on family carer emotional health, 37% a mix of positive and negative impacts, 8% no impact and 2% no impact.

Figure 1 shows the perceived impact of health and social care on domains of carers’ lives. Health and social care ‘interventions’ (i.e. treatment and replacement care) were most often associated with positive effects on carers (see Fig. 1a and b). This was particularly the case for replacement care (Fig. 1b). Here perceived impacts are very positive across all domains, with the notable exception of finances. Conversely ‘organisational changes’ (i.e. changes in timing/location of service and changes in staffing) were rarely perceived to have positive effects, with effects tending to be either negative or mixed (see Fig. 1c and d). This was particularly the case for staffing changes, where effects were very rarely perceived to be positive on balance for any domains of carers’ lives (Fig. 1d).

As Table 4 shows, this pattern of positive impacts for ‘interventions’ and mixed/negative impacts of ‘organisational changes’ was repeated across all three conditions. The negative impact of organisational changes was most pronounced in mental health. In stroke, impacts on carers were more often perceived to be mixed compared to dementia or mental health.

**Table 4:** Perceived carer effects from service delivery in dementia, mental health, and stroke

	**Emotional Health**	**Physical health**	**Finances**	**Paid work**	**Free time**
**Dementia**					
*Treatment*	Positive 71%	None 43%	None 52%	None 43%	Positive 33%
*Replacement*	Positive 62%	Positive 86%	Negative 38%	Positive 62%	Positive 76%
*Timing/location*	Positive 33%	Positive 38%	Mixed 33%	Mixed 38%	Mixed 33%
*Staffing*	Mixed 57%	Mixed 43%	None 43%	None 52%	Mixed 43%
**Mental health**					
*Treatment*	Positive 52%	Positive 52%	Positive 29%	Positive 43%	Positive 48%
*Replacement*	Positive 81%	Positive 71%	Positive 48%	Positive 62%	Positive 90%
*Timing/location*	Negative 48%	None 43%	Negative 38%	None 38%	Negative 38%
*Staffing*	Negative 48%	None 38%	None 62%	None 57%	None 38%
**Stroke**					
*Treatment*	Mixed 61%	Mixed 57%	Mixed 43%	Mixed 35%	Mixed 39%
*Replacement*	Positive 71%	Positive 74%	Mixed 48%	Positive 61%	Positive 70%
*Timing/location*	Mixed 74%	Mixed 65%	Mixed 70%	Mixed 57%	Mixed 61%
*Staffing*	Mixed 78%	Mixed 52%	None 65%	None 61%	Mixed 48%

Note: cells show the modal effect (positive/ negative/mixed/ none) and % of sample giving the modal answer. So, for example, most respondents (71%) felt dementia treatments for patients would have a positive effect on carers’ emotional health. Similarly for changes in timing and location of dementia care, the largest sub-group of respondents (33%) felt that this would have a positive effect on carers’ emotional health.

Pooling responses across all categories of health and social care and conditions (Table 5), shows which domains were most likely overall to be affected by health and social care. Overall, carers’ ‘emotional health’ was perceived most likely to be affected, with 94 % of participants responses indicating either positive, negative or mixed impacts. Conversely, finances were least likely to be perceived to be affected, with 63 % of participant responses indicating positive, negative or mixed impacts.

**Table 5:** Perceived carer effects pooled by clinical area and health and social care category (*n*=260)

	**EmotionalHealth**	**Physical health**	**Finances**	**Paid work**	**Free time**
Positive	37%	35%	16%	25%	34%
Mixed	40%	29%	30%	25%	30%
Negative	17%	13%	21%	13%	18%
None	7%	22%	33%	37%	18%

Note: this table pools results presented in Table 4 across all disease areas and intervention types to highlight the overall direction of perceived carer effects on different domains of life.

When these results are broken down by participant role it is notable that participants who were unpaid carers perceived negative effects with much greater frequency than participants who were care professionals and researchers. Specifically, carers (as compared to non-carers) perceived negative effects more frequently across all domains of life: emotional health (29 % vs. 11 %), physical health (21 % vs. 10 %), finances (29 % vs. 18 %), paid work (21 % vs. 9 %), and free time (29 % vs. 13 %).

### Consensus on including carer effects in decision-making

Overall, when pooled across condition and health and social care category, support for considering carer effects in research decisions was 81 %, support for considering carer effects in funding decisions was 81 %, and support for considering carer effects in patient care was 80 %.

Across all four clinical areas, all four service changes, and all three decision contexts (a total of 36 cells), a majority of participants agreed that carer effects should be considered in decision-making (Table 6). Consensus was achieved after round 1 for 34/36 cells (all cells for dementia and mental health and 10 of 12 cells relating to stroke). Consensus was achieved after round 2 in the 2 remaining cells (both relating to ‘staffing changes’ in stroke care) when those ‘mildly agreeing’ were included. Agreement tended to increase (towards greater consensus) between round 1 and 2 for stroke participants and was maintained at a similar (high) level in mental health and dementia.

**Table 6:** Proportion of sample ‘moderately’ or ‘strongly’ agreeing that carer impact should be considered in decision-making after 2nd round of survey

**Service change and decision context**	**Dementia**	**Mental health**	**Stroke**
**Treatment **			
*Research*	88%	90%	87%
*Funding*	94%	90%	87% ^a^
*Individual care*	81%^b^	80%	87%
**Replacement care**			
*Research*	88%	90%	91% ^a^
*Funding*	94%	90%	91% ^a^
*Individual care*	94%	90%	95% ^a^
**Timing and location**			
*Research*	81%	85%	70%
*Funding*	85%	90%	86% ^a^
*Individual care*	81% ^a^	85%	82% ^a^
**Staffing**			
*Research*	81%	80%	60%
*Funding*	94% ^a^	90%	74% ^a^
*Individual care*	94% ^a^	80%	65% ^a^

^a^ denotes greater than 10 % point increase in consensus between round 1 and 2

^b^ denotes greater than 10 % point *decrease* in consensus between round 1 and 2

In round 2, when pushed to prioritise which category of health and social care was the highest priority for inclusion of carer effects, ‘treatment’ decisions received the highest priority and considering carer effects in ‘staff changes’ received the lowest priority. For decision contexts, participants prioritised ‘patient care’ decisions most highly and ‘research’ decisions least highly.

## Discussion

This study elicited views on the impact of health and social care on unpaid carers’ lives (carer effects) and the relevance of such effects in different decision-making contexts. Expert participants perceived that healthcare and social care would affect a range of domains of carers’ lives, most commonly their emotional health. Carer effects were not universally positive, particularly for organisational changes, (changes in the timing, location of staffing of services) which were generally perceived to have negative or mixed impacts on carers’ lives. Participants viewed carer effects as important to consider in a range of decision-making contexts, most notably in decisions being made about an individual patient’s care.

This study suggests that positive effects on the carer’s emotional and physical health are likely from interventions in dementia, mental health, and stroke. This means that economic evaluations that neglect carer health may be systematically underestimating the health benefits, and therefore value, of new interventions. Conversely detrimental health effects for carers were often perceived to result from organisational changes in timing, location and staffing of health services. In these cases, economic evaluations that neglect carer health, may overestimate the benefit of the service change in the economic evaluation. Neglecting carer effects may therefore lead to ‘investment’ in organisational changes that cost-shift and are ultimately harmful to carer (and societal) health.

The finding that ‘emotional health’ is perceived to be the most likely domain to be affected is important because it underscores the need to measure carer quality of life effects in addition to time costs in economic evaluation [39, 40]. This may require a change in mindset in economic evaluation where unpaid care is not just seen only as a ‘cost’. It also highlights the need to use quality of life measures with carers that are sensitive to emotional health effects. This may point to measures of wellbeing (such as ICECAP-A) that have demonstrated sensitivity to mental ill health [41, 42] and carers experiences [34].

In this study, participants perceived carers’ finances, employment, and time, also likely to be affected by service changes. This complements recent work on the economic burden of caring [43], additionally demonstrating that service delivery may impact on carers’ financial burden. In particular, replacement care interventions were invariably perceived to positively affect free time and employment for carers. These domains should be considered on the cost side in an economic evaluation, albeit typically only when employing a societal perspective. This underscores the importance, where feasible, for a societal perspective to be used [9] to ensure the resource implications to society are properly considered. The findings from the Delphi study highlighted the fact that it could be difficult to predict the direction of carer effects. For example, replacement care may free carers up, having positive impacts on employment or free time. However, if carers bear the financial costs of respite care it may also negatively affect their personal finances.

This work adds to a body of literature advocating more consideration be given to carer effects within economic evaluation [4, 44, 45]. The widespread view, held by participants in this study, that carer effects should be considered in decision-making, is perhaps not surprising, given that many participants may have agreed to participate in this study because of an interest in unpaid carers. In view of this, participants were pushed to prioritise the *most* important contexts for considering carer wellbeing in. When this happened, participants highlighted decisions about ‘treatment’ services and decisions relating to ‘individual patient care’. On the face of it, this was unexpected, as these decisions might be thought of being more ‘patient-orientated’. This may reflect the fact that these are the decisions that participants more immediately relate to, compared with funding or research decisions. However, this finding should not take away from the consensus among participants whereby 81 % also moderately or strongly agreed that carer effects should be considered in research studies and in funding decisions. This study has identified four categories of health and social care where collection of carer data may be warranted as well as perceptions of the likely scope and direction of effects.

It is worth highlighting some of the limitations and strengths of this study. As noted earlier, a Delphi panel, by its nature, is self-selecting, so we cannot say that views expressed here are representative of care professionals or the research community more generally. In particular the unpaid carers and care professionals had participated in a previous study. This may have shaped their responses and resulted in a sample that had a particular interest in including unpaid carers in decision-making. However, this approach is likely to have reduced the rate of drop out and resulted in a sample with greater insight into carer effects and how they were likely to occur. A further point to reflect on, is that the study is limited to three conditions. Viewpoints on the relevance of including carer effects in decision-making may therefore differ for other contexts, such as end-of-life care or childhood illness where unpaid care is also likely to be important. Some participants may not have clearly understood the decision-making contexts. We briefly explained the meaning of the decision contexts in the study (e.g. for the research decisions: “Research on these interventions should include finding out how they affect carers’ lives”). However, a more in-depth explanation of funding decisions and patient care decisions might have been useful for some participants. Finally, we focused on eliciting views on broad categories of health and social care delivery, rather than specific treatments. This was done to focus on the major ways in which health and social care could impact on carers’ lives. However, a consequence of having four general categories is that there may be ambiguities about what participants associated with these categories.

A strength of this study is that we sampled participants with experience across three major conditions and in different roles. Across all there was a high degree of consistency in terms of the perceived impacts of services on carers and a high degree of consensus that these impacts should be considered in decision-making.

It is still the ‘norm’ in many countries (including the UK) to expect unpaid carers to carry much of the responsibility for patient care. Nevertheless there is much that could be done to better ensure that negative impacts of policies on carers wellbeing are minimised. Further work on this topic could focus on developing practical approaches to consider carer wellbeing in everyday care decisions. This could complement work to include carers in research studies and economic evaluation. From an economic evaluation perspective, utilising a societal perspective, and including outcome measures that encompass emotional health are important to fully capture carer effects. Other challenges exist in incorporating carer outcomes in economic evaluation, for example to include carer outcomes routinely, where primary data cannot easily be collected or in establishing how best to measuring carer outcomes where family caring networks may be complex, and extend beyond a single carer.

In conclusion, this study adds to a body of literature that highlights the importance of carer effects in economic evaluation and more generally in healthcare decision-making. It highlights the particular need to measure and value effects on carers’ emotional health and the need to use a societal perspective to avoid ‘cost-shifting’ to unpaid carers when introducing interventions and making organisational changes.

## Supplementary Information

**Additional file 1:**

**Additional file 2:**

## Data Availability

The datasets used and/or analysed during the current study are available from the corresponding author on reasonable request.
