# Supplementary material for: The scope of carer effects and their inclusion in decision-making: a UK-based Delphi study
Source: BMC Health Serv Res. 2021 Jul 29;21:752. doi: 10.1186/s12913-021-06742-4 (PMC8320027; doi:10.1186/s12913-021-06742-4)
Supplement: Supplementary file 1 — Additional file 1: [file 12913_2021_6742_MOESM1_ESM.docx]

**Appendix 1 – Text from Round 1 of Delphi survey: Mental health version (converted to MS Word)**

**Patient care and family carers’ lives: a consensus survey**

**BACKGROUND:** In this study we will ask you some questions about treatments and services (‘interventions’) a person with dementia, stroke or a mental health condition might receive. We would like to collect your views on:

- how the interventions may impact on family carers

- how any impacts should be considered in research studies and in care decisions

This information will be summarised and presented to a range of audiences (please see information sheet). We hope it will encourage researchers and decision-makers to better consider carers in their work.

**INSTRUCTIONS:** In this survey there are four intervention scenarios - one per page. The survey should take 15-30 minutes to do. Please complete all questions so that the results can properly reflect everyone's viewpoint and experience. In 3 weeks you will be sent a shorter follow-up survey to judge whether there is agreement on certain issues. Thank you very much for your time.

**NOTES:**

1. You can save your survey part way through if you want to come back to it.

2. Please think of your experiences of the interventions over time, rather than just at a particular point. If the effect of the interventions changed over time, feel free to record the different effects.

**SCENARIO A - TREATING THE PATIENT'S CONDITION**

**2. Some interventions (such as medication, psychological treatment, and lifestyle support) may prevent or treat the patient's mental health problems. Do you have personal experience of an intervention to prevent or treat the patient's mental health problems? ***

| 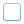 | Yes |
| --- | --- |

| 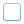 | No |
| --- | --- |

If yes, please briefly explain what intervention you have experience of and then move to the next question

|  |
| --- |

**3. If you answered 'yes' to question 2 - did this intervention impact positively and/or negatively on any of the aspects of your own life in the list below? If you answered 'no' to question 2 - would you expect this type of intervention to positively and/or negatively affect any of the following aspects of a carer's life? Please feel free to tick both positive and negative impacts if this applies. ***

|  | negative impact | positive impact | negative and positive impact | no impact |
| --- | --- | --- | --- | --- |
| Emotional health (for example: stress, anxiety or mood) | 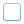 | 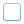 | 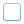 | 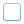 |
| Physical health (for example: fatigue, sleep or pain) | 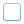 | 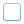 | 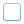 | 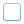 |
| Finances (for example: travel costs and other expenses) | 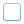 | 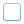 | 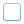 | 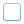 |
| Ability to do paid work | 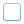 | 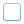 | 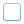 | 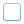 |
| Free time (for example: for leisure, family, housework, volunteering, rest) | 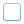 | 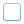 | 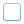 | 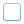 |
| Other (for example: relationships, control, sense of support, fulfilment) | 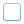 | 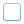 | 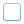 | 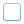 |

If 'Other' - please list any impacts

|  |
| --- |

**4. Consider interventions to prevent or treat the patient's mental health problems - do you agree or disagree with the following statements? ***

|  | strongly disagree | moderately disagree | mildly disagree | mildly agree | moderately agree | strongly agree |
| --- | --- | --- | --- | --- | --- | --- |
| Research on these interventions should include finding out how they affect carers’ lives | 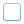 | 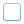 | 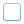 | 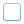 | 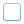 | 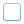 |
| Carer impacts should be considered in funding decisions | 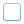 | 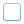 | 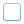 | 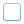 | 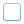 | 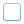 |
| Carer impacts should be considered by professionals in decisions about care | 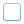 | 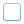 | 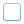 | 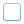 | 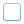 | 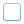 |

**INTERVENTION SCENARIO B – REPLACING FAMILY CARE**

**5. Some interventions mean that the patient will spend time with a paid health or care professional (such as a support worker, occupational therapist or care worker) supporting them in everyday activities such as leisure, shopping and travel. This means that family carers may provide less support. Do you have personal experience of an intervention replacing some of the family care? ***

| 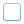 | Yes |
| --- | --- |

| 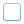 | No |
| --- | --- |

If yes, please briefly explain what intervention you have experience of and then move to the next question:

|  |
| --- |

**6. If you answered 'yes' to question 5 - did this intervention impact positively and/or negatively on any of the aspects of your own life in the list below? If you answered 'no' to question 5 - would you expect this type of intervention to positively and/or negatively affect any of the following aspects of a carer's life? Please feel free to tick both positive and negative impacts if this applies. ***

|  | negative impact | positive impact | negative and positive impact | no impact |
| --- | --- | --- | --- | --- |
| Emotional health (for example: stress, anxiety or mood) | 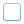 | 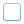 | 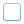 | 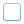 |
| Physical health (for example: fatigue, sleep or pain) | 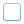 | 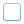 | 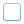 | 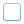 |
| Finances (for example: travel costs and other expenses) | 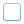 | 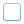 | 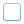 | 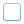 |
| Ability to do paid work | 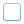 | 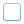 | 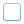 | 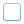 |
| Free time (for example: for leisure, family, housework, volunteering, rest) | 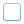 | 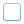 | 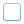 | 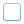 |
| Other (for example: relationships, control, sense of support, fulfilment) | 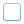 | 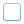 | 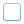 | 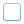 |

If 'Other' - please list any impacts

|  |
| --- |

**7. Consider interventions that replace some element of family care - do you agree or disagree with the following statements? ***

|  | strongly disagree | moderately disagree | mildly disagree | mildly agree | moderately agree | strongly agree |
| --- | --- | --- | --- | --- | --- | --- |
| Research on these interventions should include finding out how they affect carers’ lives | 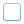 | 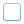 | 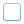 | 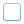 | 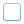 | 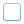 |
| Impact on carers should be considered when deciding which interventions the NHS should fund | 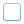 | 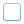 | 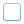 | 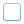 | 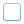 | 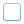 |
| Impact on carers should be considered in providing care to individual patients | 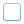 | 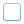 | 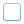 | 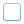 | 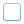 | 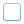 |

**SCENARIO C - CHANGING THE TIMING AND/OR LOCATION OF CARE**

**8. Some organisational changes affect the timing (e.g. time of day, frequency) or location (e.g. distance, ease of access) of patient care. Do you have personal experience of the timing and/or location of patient care being changed? ***

| 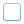 | Yes |
| --- | --- |

| 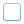 | No |
| --- | --- |

If yes, please briefly explain what intervention you have experience of and then move to the next question

|  |
| --- |

**9. If you answered 'yes' to question 8 - did this intervention impact positively and/or negatively on any of the aspects of your own life in the list below? If you answered 'no' to question 8 - would you expect this type of intervention to positively and/or negatively affect any of the following aspects of a carer's life? Please feel free to tick both positive and negative impacts if this applies. ***

|  | negative impact | positive impact | negative and positive impact | no impact |
| --- | --- | --- | --- | --- |
| Emotional health (for example: stress, anxiety or mood) | 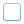 | 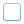 | 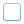 | 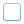 |
| Physical health (for example: fatigue, sleep or pain) | 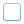 | 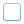 | 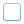 | 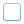 |
| Finances (for example: travel costs and other expenses) | 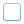 | 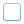 |  |  |
| Ability to do paid work |  |  |  |  |
| Free time (for example: for leisure, family, housework, volunteering, rest) |  |  |  |  |
| Other (for example: relationships, control, sense of support, fulfilment) |  |  |  |  |

If 'Other' - please list any impacts

|  |
| --- |

**10. Consider changes to the timing and/or location of patient care - do you agree or disagree with the following statements? ***

|  | strongly disagree | moderately disagree | mildly disagree | mildly agree | moderately agree | strongly agree |
| --- | --- | --- | --- | --- | --- | --- |
| Research on these interventions should include finding out how they affect carers’ lives |  |  |  |  |  |  |
| Carer impacts should be considered in funding decisions |  |  |  |  |  |  |
| Carer impacts should be considered by professionals in decisions about care |  |  |  |  |  |  |

**SCENARIO D - STAFFING CHANGES**

**11. Some organisational changes concern staffing. There may be changes, for example, to the type of care professional that sees the patient and/or how long they spend with the patient. Do you have personal experience of staffing changes with respect to patient care? ***

|  | Yes |
| --- | --- |

|  | No |
| --- | --- |

If yes, please briefly explain what intervention you have experience of and then move to the next question

|  |
| --- |

**12. If you answered 'yes' to question 11 - did this intervention impact positively and/or negatively on any of the aspects of your own life in the list below? If you answered 'no' to question 11 - would you expect this type of intervention to positively and/or negatively affect any of the following aspects of a carer's life? Please feel free to tick both positive and negative impacts if this applies. ***

|  | negative impact | positive impact | negative and positive impact | no impact |
| --- | --- | --- | --- | --- |
| Emotional health (for example: stress, anxiety or mood) |  |  |  |  |
| Physical health (for example: fatigue, sleep or pain) |  |  |  |  |
| Finances (for example: travel costs and other expenses) |  |  |  |  |
| Ability to do paid work |  |  |  |  |
| Free time (for example: for leisure, family, housework, volunteering, rest) |  |  |  |  |
| Other (for example: relationships, control, sense of support, fulfilment) |  |  |  |  |

If 'Other' - please list any impacts

|  |
| --- |

**13. Consider organisational changes that affect staff involved in patient care - do you agree or disagree with the following statements? ***

|  | strongly disagree | moderately disagree | mildly disagree | mildly agree | moderately agree | strongly agree |
| --- | --- | --- | --- | --- | --- | --- |
| Research on these interventions should include finding out how they affect carers’ lives |  |  |  |  |  |  |
| Carer impacts should be considered in funding decisions |  |  |  |  |  |  |
| Carer impacts should be considered by professionals in decisions about care |  |  |  |  |  |  |
