# Supplementary material for: The scope of carer effects and their inclusion in decision-making: a UK-based Delphi study
Source: BMC Health Serv Res. 2021 Jul 29;21:752. doi: 10.1186/s12913-021-06742-4 (PMC8320027; doi:10.1186/s12913-021-06742-4)
Supplement: Supplementary file 2 — Additional file 2: [file 12913_2021_6742_MOESM2_ESM.docx]

**Appendix 2: Example feedback sheet for Round 2**

#### **Name: JOE BLOGGS**

#### Delphi study ID: MC01

This is a copy of the information collected from Round 1 of the Delphi Survey. Our target was to get at least 20 respondents with expertise in each of the following areas: dementia, mental health and stroke. We were grateful to you that we achieved this. The 65 respondents included a good spread across the three different roles, with the following taking part:

- 21 family carers (including 9 in mental health)
- 21 care professionals (including 7 in mental health)
- 23 researchers/academics (including 5 in mental health)

The remainder of this report summarises key information from round 1 that we would like you to consider when providing your responses in round 2:

- Page 2 – Scenario A – treating and preventing mental health problems
- Page 3 – Scenario B – replacing elements of family care
- Page 4 – Scenario C – changes in the location and timing of service users’ care
- Page 5 – Scenario D – changes in staffing related to service users’ care

**For round 2**

The aim of round 2 is to see if a consensus emerges on the circumstances when impacts on family carers should be taken into account, alongside the service user, in decision-making. This document presents your answers alongside the answers of the rest of the 20 respondents with expertise in mental health. We would like you to consider all the responses and judge whether you wish to stand by or change your view. Either is fine! Please review the document as you re-complete the online survey.

**Many thanks for your input and support in this research project**

**SECTION A - TREATING THE SERVICE USER’S CONDITION**

**When completing Scenario A and the ranking tasks please consider the information below.**

All 21 respondents reported having personal or professional experience of services to prevent or treat service users' mental health problems.

***Should impacts on family carers be considered in decisions about preventing and treating service users’ mental health problems?***

- 90% of respondents moderately or strongly agreed that family carers should be considered in research studies. YOUR ANSWER WAS STRONGLY AGREE
- 86% of respondents moderately or strongly agreed that family carers should be considered in government funding decisions. YOUR ANSWER WAS STRONGLY AGREE
- 81% of respondents moderately or strongly agreed that family carers should be considered in decisions about care for individual service users. YOUR ANSWER WAS STRONGLY AGREE

**Figure 1: Proportion of respondents (n=21) agreeing that family carers should be considered in decisions about preventing and treating service users’ mental health problems**

Services to prevent or treat service users’ mental health problems were often perceived to impact positively on carers’ lives (see figure 2), particularly in terms of emotional and physical health. However negative or mixed effects on carers were also perceived.

**Figure 2: Proportion of respondents (n=21) perceiving negative and positive impacts on family carers from services to prevent and treat service users’ mental health problems**

**SECTION B - REPLACING FAMILY CARE**

**When completing Scenario B and the ranking tasks please consider the information below.**

17 out of 21 respondents reported having personal or professional experience of services that replace some elements of family care.

***Should impacts on family carers be considered in decisions about services that replace some elements of family care?***

- 90% of respondents moderately or strongly agreed that family carers should be considered in research studies. YOUR ANSWER WAS STRONGLY AGREE
- 90% of respondents moderately or strongly agreed that family carers should be considered in government funding decisions. YOUR ANSWER WAS STRONGLY AGREE
- 86% of respondents moderately or strongly agreed that family carers should be considered in decisions about care for individual service users. YOUR ANSWER WAS STRONGLY AGREE

**Figure 3: Proportion of respondents (n=21) agreeing that family carers should be considered in decisions about services that replace some elements of family care**

Services that replace some element of family care were invariably perceived to impact positively on carers’ lives, particularly in terms of freeing up carers’ time (90%), but also in terms of physical and emotional health (both >70%).

**Figure 4: Proportion of respondents (n=21) perceiving negative and positive impacts on family carers from services to replace some element of family care**

**SECTION C – CHANGING THE TIMING OR LOCATION OF CARE**

**When completing Scenario C and the ranking tasks please consider the information below.**

15 out of 21 respondents reported having personal or professional experience of changes in the timing and location of service users’ care.

***Should impacts on family carers be considered in decisions about the timing and location of service users’ care?***

- 86% of respondents moderately or strongly agreed that family carers should be considered in research studies. YOUR ANSWER WAS STRONGLY AGREE
- 86% of respondents moderately or strongly agreed that family carers should be considered in government funding decisions. YOUR ANSWER WAS STRONGLY AGREE
- 86% of respondents moderately or strongly agreed that family carers should be considered in decisions about care for individual service users. YOUR ANSWER WAS STRONGLY AGREE

**Figure 5: Proportion of respondents (n=21) agreeing that family carers should be considered in timing and location changes related to service users’ care**

Timing and location changes were less often perceived to impact positively on family carers (see figure 6), with many respondents highlighting negative impacts (particularly in emotional health) or no impacts.

**Figure 6: Proportion of respondents (n=21) perceiving negative and positive impacts on family carers from timing and location changes related to service users’ care**

**SECTION D – STAFFING CHANGES RELATED TO SERVICE USERS’ CARE**

**When completing Scenario D and the ranking tasks please consider the information below.**

18 out of 21 respondents reported having personal or professional experience of staffing changes related to service users’ care.

***Should impacts on family carers be considered in decisions about staffing changes related to service users’ care?***

- 86% of respondents moderately or strongly agreed that family carers should be considered in research studies. YOUR ANSWER WAS STRONGLY AGREE
- 86% of respondents moderately or strongly agreed that family carers should be considered in government funding decisions. YOUR ANSWER WAS STRONGLY AGREE
- 81% of respondents moderately or strongly agreed that family carers should be considered in decisions about care for individual service users. YOUR ANSWER WAS STRONGLY AGREE

**Figure 7: Proportion of respondents (n=21) agreeing that family carers should be considered in staffing changes related to service users’ care**

Staffing changes were very rarely perceived to impact positively on family carers (see figure 8), with over half of respondents either perceiving negative or mixed impacts on family carers’ emotional and physical health and free time. Most respondents did not perceive impacts on finances or ability to work.

**Figure 8: Proportion of respondents (n=21) perceiving negative and positive impacts on family carers from staffing changes related to service users’ care**
